# Editorial: Novel techniques and technologies in spine surgery: New approaches for the traumatic, oncologic, and aging spine

**DOI:** 10.3389/fsurg.2023.1155636

**Published:** 2023-02-20

**Authors:** Mauro Palmieri, Alessandro Frati, Giancarlo D’Andrea, Antonio Santoro, Maurizio Salvati, Alessandro Pesce

**Affiliations:** ^1^ Neurosurgery Division, Università “La Sapienza” di Roma, Roma, Italy; ^2^ Neurosurgery, IRCCS-“Neuromed”, Pozzilli, Italy; ^3^ Neurosurgery Division, “Spaziani” University Hospital, Frosinone, Italy; ^4^ Department of Neurosurgery, Policlinico “Tor Vergata”, University of Rome “Tor Vergata”, Rome, Italy; ^5^ Neurosurgery Division, A.O. “Santa Maria Goretti”, Latina, Italy

**Keywords:** spine, spine surgery, spinal instrumentation, trauma, degenerative disease

**Editorial on the Research Topic** Novel techniques and technologies in spine surgery: New approaches for the traumatic, oncologic, and aging spine

Spine surgery is indicated for several medical conditions with different pathogenesis and due to its variability and complexity, it represents a field in continuous development. First, it is not possible to ensemble all pathologies in the same group, since traumatic spine conditions are very different from oncological diseases and from degenerative conditions due to aging. More specifically, spine surgeons must confront themselves with different scenarios according to the kind of spinal conditions that they have to confront; for instance, it is not the same to confront with a traumatic vertebral fracture and with a pathological one caused by a secondary localization of a solid tumor or by osteoporosis. Therefore, this great variety in diagnostic and treatment protocols leads to the research of more accurate and less invasive tools to guarantee patient's health with minimum amount of discomfort and complications, especially with the development of new surgical techniques and technologies.

The aim of the special issue entitled “Novel techniques and technologies in Spine Surgery: New approaches for the traumatic, oncologic, and aging Spine” is to present updated evidence in all the three big fields of spine surgery: traumatic, oncologic, and degenerative diseases, with a special focus on surgical technique and application of new technologies.

Regarding degenerative spine disease related to aging, Zhang et al. in their paper entitled “*Comparison of posterior decompression techniques and conventional laminectomy for lumbar spinal stenosis*” focus on one of the most common degenerative diseases that affects lumbar spine, which is lumbar stenosis. More specifically, they report a meta-analysis conducted on 14 trials comprehending 1,106 participants, in which the conventional laminectomy as standard decompression technique is compared to bilateral laminotomy. The results showed that in terms of functional recovery, postoperative stability, and postoperative rehabilitation outcomes bilateral laminotomy appears to provide better outcomes than laminectomy, even though none of the included trials reported long-term results.

Moving from lumbar to cervical spine, Mu et al. in their paper entitled “*Anterior cervical discectomy and fusion with zero-profile versus stand-alone cages for two-level cervical spondylosis: A retrospective cohort study*” focus on another common degenerative disease of the spine: cervical spondylosis.” In this retrospective cohort study, the Authors compare the application of two different instrumentations for Anterior Cervical Discectomy and Fusion (ACDF). Several papers compared the conventional use of stand-alone cages (ST) with zero-profile (ZP) cages for single level ACDF. Even though the results in term of fusion rate and incidence of dysphagia are similar, up to date there are no large studies regarding the use of these two types of cervical cages in multilevel cervical spondylosis. In this paper it is reported that both ZP and ST both represent a good alternative for the treatment of this conditions, with ZP allowing to achieve better lower loss of disc height, which leads to better outcomes in restoring the physiological cervical spine curvature.

Remaining on the topic of cervical spine degenerative conditions, Ching-Li et al. in their paper entitled “*The surgical strategy for multilevel massive ossification of the posterior longitudinal ligaments*” report the result of a retrospective series of patients treated for multilevel ossification of the posterior longitudinal ligaments (OPLL). The gold standard for the treatment of OPLL has not been identified yet and it is still debated if a combined anterior and posterior approach is better than only anterior or posterior techniques. Of the 55 patients included in the final cohort, 40 have been treated with posterior laminectomy and ACDF, while the remaining 15 have neem treated with cervical laminectomy and posterior fixation. The Authors reported that patient treated with both anterior and posterior approaches reach better outcomes in terms of restoring sagittal lordosis and long-term pain, while no differences in the two groups has been highlighted in term of functional outcome. Moreover, they remind that in the decisional process regarding which anterior approach is better (discectomy versus corpectomy), clinical and radiological features of each patient must be taken in account.

In the last paper of this special issue, the focus is moved from degenerative to oncological diseases. Marengo et al. in their paper entitled “*3D-printed guides for cervical pedicle screw placement in primary spine tumor: Case report and technical description*” report the application of a novel technology in spine surgery, which is the 3D-printed guides for spine instrumentations. In this paper, the Authors focus on one of the major issues in spine surgery, which is the necessity of reaching good fusion rates while lowering the rates of mispositioning, screw loosening and surgical complications. This issue is particularly relevant in conditions that cause degeneration of patient's bone, like oncological diseases. In this paper, the use of 3D-printed guides for cervical screws positioning in a patient affected by a craniovertebral junction chordoma is described with good outcomes in terms of functional and radiological status. Even though just one case is reported, the focus on the technique is interesting, since it suggests a valuable solution for overcoming instrumentation failure by preparing a real tailored approach.

As Guest Editors of this special issue, we believe that the readership of “Frontiers in Surgery” will find interesting and novel information regarding the treatment of spinal conditions.

